# The Effect of Different Compositions and Concentrations of Etidronate-Containing Irrigants on the Antibacterial Activity of Sodium Hypochlorite against *Enterococcus faecalis* and *Candida albicans*

**DOI:** 10.3390/dj12030046

**Published:** 2024-02-21

**Authors:** Nina Novozhilova, Ksenia Babina, Maria Polyakova, Inna Sokhova, Valeria Sherstneva, Alexandr Zaytsev, Irina Makeeva, Anna Mikheikina

**Affiliations:** 1 Department of Therapeutic Dentistry, I.M. Sechenov First Moscow State Medical University (Sechenov University), 119991 Moscow, Russia; novozhilova_n_e@staff.sechenov.ru (N.N.); babina_k_s@staff.sechenov.ru (K.B.); polyakova_m_a_1@staff.sechenov.ru (M.P.); sokhova_i_a@staff.sechenov.ru (I.S.); valeria_sherstneva@mail.ru (V.S.); makeeva_i_m@staff.sechenov.ru (I.M.); 2 Institute of Linguistics and Intercultural Communication, I.M. Sechenov First Moscow State Medical University (Sechenov University), 119991 Moscow, Russia; zaytsev_a_b@staff.sechenov.ru

**Keywords:** *C. albicans*, *E. faecalis*, etidronic acid, root canal irrigants, sodium hypochlorite

## Abstract

We assessed the effect of different compositions and concentrations of two etidronate-containing irrigants on the antibacterial activity of sodium hypochlorite (SH) against *Enterococcus faecalis* and *Candida albicans* in vitro. Pure cultures of *C. albicans* and *E. faecalis* were isolated from root canal samples. The disc diffusion method was used to compare the antibacterial effect of pure SH and SH mixed with 9%, 15%, and 18% etidronate of two manufactures (dual rinse (DR); IsraDent (ID)) and EDTA. The pH and temperature of the solutions were measured immediately after mixing and within 40 min. The ANOVA revealed a significant influence of the type of irrigating solution on the *C. albicans* and *E. faecalis* inhibition zone diameters that ranged from 6.6 to 51.6 mm and from 6.4 to 12.4 mm, respectively. SH with DR 9% exhibited the highest effect against *C. albicans*. The antifungal activity of the other irrigants was SH = SH + DR15% = SH + DR18% = SH + ID9% > SH + EDTA > SH + ID15% > SH + ID18%. No significant differences in the anti-E. faecalis effect were revealed between the tested solutions except for the mixtures of SH and 15% and 18% ID, which exhibited no antiseptic effect. There was a strong positive correlation between antiseptic activity against both microorganisms and the pH values of the tested solutions. In conclusion, most etidronate formulations did not significantly hamper sodium hypochlorite activity against *C. albicans* and *E. faecalis*. The effect was concentration- and manufacturer-dependent.

## 1. Introduction

The purpose of endodontic treatment is the disinfection and obturation of the root canal system [1]. Disinfection is achieved using root canal instrumentation and irrigation. Root canal instrumentation is aimed at removing pulp tissues and infected dentin from root canal walls and creating a shape and taper favouring three-dimensional fillings. At the same time, using the hand or rotary instruments results in the formation of a layer of organic and inorganic material (the smear layer) on root canal walls [2]. The smear layer occludes the lumens of dentinal tubules and accessory canals, thus preventing the penetration of intracanal medicaments and interfering with the adhesion of endodontic sealers to dentin [3,4,5]. Therefore, the removal of this layer is indicated for the disinfection of the root canal system and better adaptation of materials to the canal walls [6,7].

Several solutions and their combinations have been proposed for root canal irrigation. It is well established that sodium hypochlorite (SH) provides not only antimicrobial but also tissue-dissolving activity, which, however, is limited to the organic components of the smear layer [8]. Thus, the supplementary use of demineralizing (chelating) agents is recommended during root canal treatment [9]. The most common endodontic chelating agent is ethylenediaminetetraacetic acid (EDTA) [10,11]. EDTA reacts with calcium ions in dentin and forms soluble calcium chelates, which are further easily eliminated from the root canal system. To potentiate smear layer removal, one may use ultrasonic [4,12] or sonic activation [13], negative-pressure irrigation [14], laser activation [15], and manual dynamic agitation [16].

The sequential use of EDTA and SH is widely accepted [17,18,19,20,21]; however, this irrigation protocol has some limitations. First, a chemical reaction occurring in a mixture of these solutions results in the loss of the antimicrobial and tissue-dissolving properties of SH due to the loss of free available chlorine [11,22,23]. Second, EDTA decreases dentin flexural strength and denatures collagen fibres [24,25], decreasing the adhesion of obturating material to the root canal walls [22,26,27]. Similar effects have been demonstrated by citric [28] and maleic [29] acids when combined with SH.

To address these limitations, a combination of SH and a weak chelator (such as tetrasodium EDTA [30], clodronate [31], or etidronic acid [23]) has been advocated for continuous irrigation throughout root canal instrumentation. A mixture of EDTA in the form of tetrasodium salt (Na4EDTA) with SH, when used in continuous chelation, showed shorter use life (according to the content of free available chlorine) compared with its mixture with clodronate and etidronate [32]. Despite the promising results of clodronate as a potential endodontic chelator, currently, there are no commercially available irrigants based on this agent. Etidronic acid (1-Hydroxyethylidene-1, 1-Bisphosphonate, HEBP, or HEDP) is a systemic medicament used in the treatment of osteoporosis and Paget’s disease, as it decreases bone resorption [33]. In endodontics, etidronate is used in the form of tetrasodium salt (Na4etidronate). The effect of etidronate on the antimicrobial activity of SH has been investigated in a number of studies [34,35,36,37]. According to earlier investigations, despite the fact that mixing SH with HEDP resulted in a reduction in free available chlorine after one hour, its antibacterial effects were preserved, making this mixture theoretically suitable for chemomechanical preparation [23,31,37,38]. However, there is a paucity of literature on the effect of different concentrations of etidronate on the antimicrobial activity of the mixture.

Traditionally, the antibacterial effects of endodontic irrigants are studied using *Enterococcus faecalis* species, as they have been frequently detected in teeth with apical periodontitis [39,40,41]. At the same time, *Candida albicans* is the most prevalent species of fungi associated with persistent endodontic infections [42]. To the best of our knowledge, the literature on the effect of a SH–etidronate mixture on *Candida albicans* is scarce [43,44,45].

Therefore, our study aimed to assess the effect of different compositions and concentrations of etidronate-containing irrigants on the antibacterial activity of SH against *Enterococcus faecalis* and *Candida albicans*.

## 2. Materials and Methods

The study protocol was approved by the Ethics Committee of Sechenov University, Moscow, Russia (No. 22-02).

### 2.1. Irrigant Preparation, and pH and Temperature Measurement

For this study, the following irrigating solutions were evaluated:

- SH—sodium hypochlorite (“Hypochloran-3”, Omegadent, Moscow, Russia);
- SH + EDTA—sodium hypochlorite (“Hypochloran-3”, Omegadent, Moscow, Russia) mixed with 17% EDTA (“MD-Cleanser”, Metabiomed, Cheongju, Republic of Korea);
- SH + DR—sodium hypochlorite (“Hypochloran-3”, Omegadent, Moscow, Russia) mixed with Dual Rinse^®^ HEDP (Medcem, Weinfelden, Switzerland);
- SH + ID—sodium hypochlorite (“Hypochloran-3”, Omegadent, Moscow, Russia mixed with Isradent^®^ HEDP (“HEBP Etidronic acid”, Isradent, Tyumen, Russia);
- DW—distilled water (control).

All irrigants were freshly mixed using glass stirring rods in sterile glass containers. SH and EDTA were mixed at a ratio of 1:1, in an amount of 5 mL each. SH and etidronate mixtures were prepared in accordance with the manufacturer’s instructions. SH + DR mixtures were prepared by adding 0.9 (the mean content per capsule), 1.5, or 1.8 g of Dual Rinse^®^ HEDP powder to 10 mL of SH to obtain 9%, 15%, or 18% solutions, respectively. SH + ID mixtures were prepared by adding 0.9 (mean content per ampul), 1.5, or 1.8 g of Isradent^®^ HEDP powder to 10 mL of SH to obtain 9%, 15%, or 18% solutions, respectively. The weight of the HEDP powder was adjusted using a hermetic precision electronic balance (Sartorius AG, Göttingen, Germany).

The pH and temperature of the solutions was measured at room temperature immediately after mixing, at 5, 10, 15, 20, 30, and 40 min, using a calibrated pH meter and thermometer (MILWAUKEE PH56 PRO, Rocky Mount, NC, USA).

### 2.2. Antiseptic Effect Assessment

The samples were taken from the root canal (upper canine with chronic apical periodontitis) of the consenting patient using a sterile paper point by inserting it into the full length of the root canal and retaining it in that position for 60 s. Before taking the samples, the tooth was isolated with a rubber dam and rinsed with 0.2% chlorhexidine solution. A sterile bur was used to create the access cavity. Four sterile paper points (MTwo Absorbent Paper Points, VDW GmbH, Munich, Germany) were introduced into the root canal and kept in place for 60 s to absorb the exudate.

#### 2.2.1. *Candida albicans*

##### Identification of a Pure Culture

After collection, the samples (two paper points) were immediately streaked on Sabouraud chloramphenicol agar (Obolensk, Russia) and incubated at 35–37 °C for 24–48 h. The colonies were screened preliminarily via morphological observation and Gram staining. Suspected creamy white, shiny, convex dome-shaped, glabrous, smooth-edged colonies were selected for culture purification. After Gram staining, a microscopic examination of the selected colonies showed blue-purple, round, or oval budding yeast cells. Differential culture media (*Candida* Chromogenic agar, Conda, Spain) was used for the identification of *Candida albicans*.

##### Antiseptic Susceptibility Testing

Standardised suspensions were then prepared and adjusted to an optical density of 0.5 at 535 nm using a spectrophotometer (Densi-La-Meter, Erba Lachema s.r.o., Brno, Czech Republic). The antiseptic effect was tested using Mueller–Hinton Agar with glycose and methylene blue. The Petri dishes were spread plated with 0.1 mL of standardised suspension. Five Petri dishes were not spread-plated (negative control). The discs soaked in the tested solutions (*n* = 5 for each solution) were placed in the Petri plates and incubated at 37 °C. The inhibition zone diameters (IZDs) were measured following 24 h of incubation.

#### 2.2.2. *Enterococcus faecalis*

##### Identification of a Pure Culture

After collection, the samples (two paper points) were immediately streaked on 5% blood agar (HiMedia Laboratories Pvt. Limited, Mumbai, India) and incubated at 35–37 °C for 18–20 h. Suspected semitransparent colonies measuring 1–2 mm in diameter were selected for culture purification. The colonies were screened via microscopic examination after Gram staining and peroxidase activity assessment. The colonies with catalase-negative, Gram-negative cocci arranged in pairs, singly, or in short chains were selected. Pure colonies were identified using the EN-COCCUS test (Erba Lachema s.r.o., Brno, Czech Republic) and Multiscan FC Microplate Photometer (Thermo Fisher Scientific Inc., Waltham, MA, USA).

##### Antiseptic Susceptibility Testing

Standardised suspensions were then prepared and adjusted to an optical density of 0.5 at 535 nm using a spectrophotometer (Densi-La-Meter, Erba Lachema s.r.o., Brno, Czech Republic). The antiseptic effect was tested using Mueller–Hinton Agar with 5% blood. The Petri dishes were spread-plated with 0.1 mL of standardised suspension. Five Petri dishes were not spread-plated (negative control). The discs soaked in the tested solutions (*n* = 5 for each solution) were placed in the Petri plates and incubated at 37 °C. The IZDs were observed following 24 h of incubation.

### 2.3. Statistical Analyses

Data entry was completed in an MS Excel file (Excel for Mac version 16.79.1 (23111614), Microsoft corp., Mountain View, CA, USA). The data were exported into CSV file format, and were then used for data analysis in R (version 4.2.3 (15 March 2023), R Development Core Team, Columbia university, New York, NY, USA) using the following packages: “doBy”, “rstatix”, “tidyverse”, “ggpubr”, “stats”, and “PerformanceAnalytics”.

Data were presented as means and standard deviations, and medians and interquartile ranges. The normality and sphericity of distribution were assessed with Shapiro–Wilk and Levene’s tests, respectively. As the assumptions of normality and sphericity were met, ANOVA was performed followed by a post hoc Tukey test with adjustment for multiple comparisons. Pearson’s correlation coefficient was calculated to reveal the pair-wise correlation between the pH of the solutions immediately after mixing and the mean IZDs for *C. albicans* and *E. faecalis*.

## 3. Results

### 3.1. pH and Temperature Measurements of the Tested Solutions

**Figure 1:**
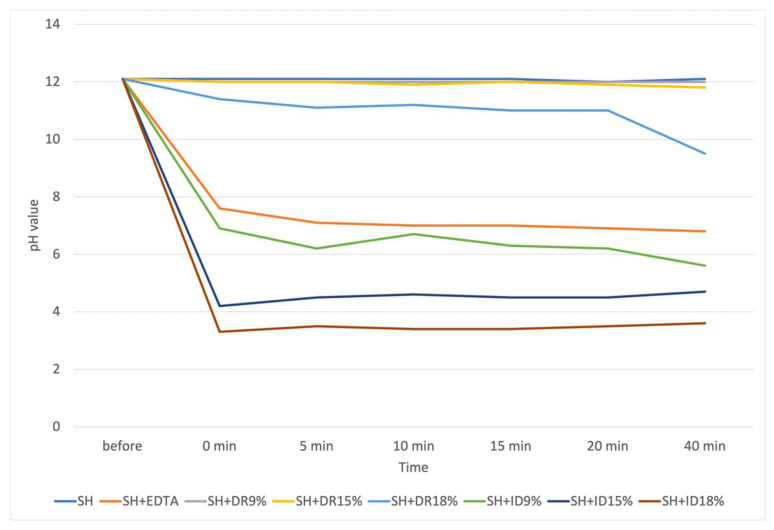
PH dynamics of pure SH and SH mixed with different irrigating solutions.

The pH dynamics are shown in Figure 1. Pure SH and the SH + DR9% and SH + DR15% mixtures maintained a pH level of around 12 throughout the entire experiment (40 min).

In contrast, the SH + EDTA mixture demonstrated an abrupt drop to a pH of 7.6 immediately after mixing. The SH + DR18% mixture maintained a pH of 11 or above for 20 min, which then fell to pH 9.5 at 40 min. The SH + ID9%, SH + ID15%, and SH + 18% mixtures all showed dramatic falls in pH, dropping to pH 6.9, 4.2, and pH 3.3, respectively, by 1 min.

Large pH falls in the SH + EDTA mixture and SH + ID mixtures (all concentrations) were accompanied by a rise in temperature. The temperature in these solutions rose from the baseline of 23–24 °C to 28–29 °C immediately after mixing. Pure sodium hypochlorite and SD + DR mixtures (all concentrations) exhibited no considerable temperature changes.

### 3.2. Antiseptic Effect Assessment

The disc diffusion method was used to assess the susceptibility of the tested microorganisms. The results are presented in Table 1. All negative controls showed no bacterial growth. No inhibition of bacterial growth was observed in the DW group (positive control).

#### 3.2.1. Antiseptic Effect of the Tested Solutions against *C. albicans*

The ANOVA revealed a significant influence of the type of irrigating solution on the IZDs that ranged from 6.6 (0.9) to 51.6 (2.1) mm (*p* < 0.001).

The application of disks soaked in 3% sodium hypochlorite resulted in a mean IZD of 46.4 (2.8) mm. Mixing with EDTA significantly decreased the effect of sodium hypochlorite against *C. albicans* (*p* < 0.001). Disks impregnated with mixtures of sodium hypochlorite and different HEDP solutions produced inhibition zones of different diameters. The effect of the SH + HEDP mixtures was concentration- and manufacturer-dependent. All HEDP formulations did not significantly decrease the activity of SH + ID15% and SH + ID18%. The antiseptic effect exhibited by the SH + DH9% mixture was significantly greater than those exhibited by the other irrigating solutions. The antiseptic effect exhibited by the SH + ID15% mixture was significantly smaller than those exhibited by the other irrigating solutions. The SH + ID18% mixture completely inactivated the antiseptic activity of the former.

#### 3.2.2. Antiseptic Effect of the Tested Solutions against *E. faecalis*

The ANOVA revealed a significant influence of the type of irrigating solution on IZDs that ranged from 6.4 (0.5) to 12.4 (0.9) mm (*p* < 0.001). No significant differences were revealed between the tested solutions except for the SH + 15%ID and SH + 18%ID mixtures, which exhibited no antiseptic effect on E. faecalis. The rest of the irrigating solutions did not inactivate the antiseptic activity of sodium hypochlorite.

### 3.3. Correlation Analysis

**Figure 2:**
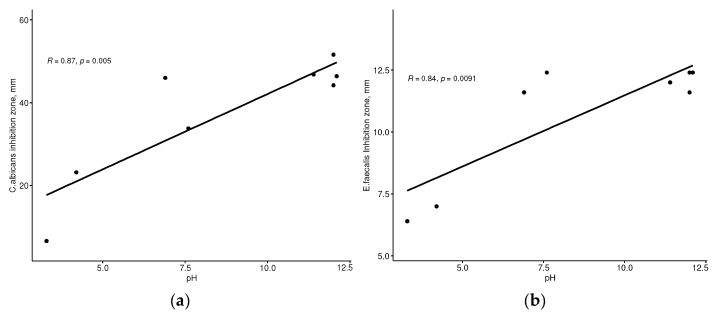
The correlation between pH values and inhibition zone diameters for *C. albicans* (**a**) and *E. faecalis (***b**).

Figure 2 shows the results of the correlation analysis of the IZDs and pH of the irrigants. We found a strong positive correlation between the antiseptic activity against *C. albicans* (r = 0.87, *p* = 0.004972) and *E. faecalis* (r = 0.84, *p* = 0.009063), and the pH values of the tested solutions.

## 4. Discussion

This study compared the effect of EDTA and HEDP on the antiseptic activity of sodium hypochlorite against *C. albicans* and *E. faecalis*. We found that, in contrast to EDTA, all HEDP formulations except those with 15% and 18% ID did not significantly decrease the activity of SH against *C. albicans*. The effect of the tested solutions on *E. faecalis* was minor; SH + ID15% and SH + ID18% showed no effect at all.

There is a relatively small body of literature that is concerned with the effect of endodontic irrigants containing HEDP on *C. albicans*. The existing studies vary in experimental design, and their results are difficult to compare. Tartari et al. investigated the effects of combinations of several irrigants on the adhesion of *C. albicans* to dentin disks [44]. In their study, the SH2.5% and SH2.5% + HEDP9% mixtures demonstrated significantly weaker effects than SH5% + HEDP18% and SH2.5% + EDTA mixtures. However, their study does not provide information on the antifungal activity of the investigated solutions.

Karale et al. tested the antimicrobial effect of various irrigating solutions against *C. albicans* in the presence and in the absence of dentin powder. Suspensions containing dentin powder, irrigating solutions, and *C. albicans* were submitted to serial dilution, and colony-forming units were counted. The authors found that in the absence of dentin powder, SH showed a significant antifungal effect, while 18% HEDP solution provided no antifungal effect [45]. Unfortunately, their study did not assess antifungal effect of the SH + HEDP mixture.

In our study, the effect of SH + HEDP mixtures depended on the HEDP concentration and formulations. SH + HEDP9% solutions exhibited a significantly stronger antifungal effect when compared to higher concentrations of HEDP produced by the same manufacturer. However, all DR mixtures were significantly more effective than ID mixtures of similar concentrations. The antiseptic effect exhibited by the SH + DR9% mixture was significantly greater than those exhibited by the other irrigating solutions, including pure SH. EDTA significantly decreased the antifungal activity of SH; this mixture showed worse results than all DR formulations and ID9%.

The only report partially comparable to ours was that of Alshanta et al., who tested the effect of three endodontic irrigants (3% SH, 3% SH followed by 17% EDTA, or SH3% + HEDP9%) against *C. albicans* [43]. All three treatments showed a similar positive antimicrobial effect on *C. albicans*. There were no significant differences between pure SH and the SH + HEDP mixture. However, their results are still difficult to compare with ours as the authors used *C. albicans* and *E. faecalis* mono- and dual-species biofilms and assessed the antibacterial activity of the tested solutions with real-time quantitative PCR. Noteworthily, in their study, *C. albicans* was more sensitive to all tested irrigants compared with *E. faecalis* [43]. These results are in agreement with the results of our study. The IZDs for all tested irrigants were smaller in *E. faecalis* cultures than those in *C. albicans* cultures.

Regarding the antibacterial activity against *E. faecalis*, we found no significant differences among pure SH, SH + EDTA, SH + DR (9%, 15%, and 18%), and SH + ID9%. No antiseptic effect was demonstrated by 15% and 18% ID. Several studies have investigated the effect of HEDP 9% on the anti-*E. faecalis* activity of SH and found that HEDP did not hamper it. Morago et al. evaluated the influence of the smear layer on the antimicrobial activity of SH2.5% alone and combined with HEDP 9% against *E. feacalis* in dentinal tubules. They measured the percentage of dead cells using confocal laser scanning microscopy and the live/dead technique and found that SH2.5% alone and combined with HEDP 9% were equally effective against *E. feacalis* in the dentinal tubules [37]. Campello et al. evaluated bacterial reduction promoted via chemomechanical root canal preparation in extracted human teeth. They found that SH2.5% + HEDP9% was significantly more effective than SH alone regarding intracanal bacteria reduction assessed via quantitative PCR [35]. In a study by Arias-Moliz, *E. faecalis* growing in biofilms and in dentinal tubules were eradicated by SH 2.5% alone and associated with HEDP 9% after 10 min of contact time. There was no statistical difference between these solutions [46]. Another study conducted by the same authors showed that SH 2.5% alone and combined with HEDP 9% were the only irrigants capable of killing over 85% of *E. faecalis* biofilms [47]. These two studies used confocal laser scanning microscopy and the live/dead technique. The results of the in vitro studies were confirmed in a clinical trial by Ballal et al., who compared the effect of irrigation with the SH2,5% + HEDP9% mixture and that of irrigation with SH2.5%. The use of SH alone resulted in 40% of root canals being free of culturable microorganisms, while the use of SH + HEDP9% resulted in 50% of root canals being free of microorganisms. Differences were insignificant; therefore, it may be concluded that the antibacterial effect of SH in vivo was not altered by HEDP [48].

In other studies, the effect of HEDP on the anti-*E. faecalis* activity of SH has been compared with that of EDTA. Alshanta et al. found that for *E. faecalis*, the immediate killing effect of SH alone, an SH3% + HEDP9% mixture, and SH3% + EDTA17% treatments resulted in ≥98% bacterial reduction, with no statistical differences between the three treatments [43]. Similarly, in a study by Pedrinha et al., SH 5% followed by an EDTA and SH5% + HEDP18% mixture demonstrated a comparable reduction in viable bacteria counts inside the main root canals and in the dentinal tubules after chemomechanical preparation according to confocal laser scanning microscopy [49]. These results are in accordance with our observations, which showed that there were no significant differences between the anti-*E. faecalis* effects of pure SH, SH + EDTA, SH + DR (9%, 15%, and 18%), and SH + ID9% mixtures.

However, these findings differ from those of other studies, which have suggested that the anti-*E. faecalis* activity of SH + HEDP is superior to that of SH + EDTA [50,51,52]. Neelakantan et al. assessed the influence of different irrigation protocols on mature *E. faecalis* biofilms using confocal laser scanning microscopy and the live/dead technique. They found a significantly greater bacterial reduction in the SH6% + HEDP18% group than in the SH6% + EDTA group [50]. In the experimental biofilm model adapted by Álvarez-Sagües et al., the use of 9% HEDP dissolved in 5.25% SH was more effective than the use of SH5.25% + EDTA17% for the elimination of the remaining *E. faecalis.* The effect was assessed using colony-forming unit counts and SEM [51]. Similar results were obtained in a study by Giardino et al., in which residual bacterial viability (assessed with confocal laser microscopy) was significantly lower after irrigation with SH5% + HEDP18% than that after irrigation with SH + EDTA [52]. All in all, it may be concluded that the anti-*E. faecalis* effect of the SH + HEDP mixture was similar to or greater than the effect of pure SH and SH + EDTA.

Another issue that is important to discuss is the mechanism that leads to the loss of SH activity. Antimicrobial and proteolytic effects of SH are produced by free available chlorine, which consists of hypochlorite (OCl−) and hypochlorous acid (HOCl) [11,53]. The free available chlorine concentration in SH decreases over time, with exposure to light or heat, and on contact with various substances, including EDTA, which is commonly used with SH during endodontic irrigation [53]. The reaction between these two solutions results in the rapid loss of free available chlorine [23,54], rendering SH ineffective on bacteria and necrotic tissue. Weak chelators such as HEDP can be mixed with SH without the considerable loss of its free available chlorine in the short term, thus preserving its activity [23,30,31,32,38]. In contrast to EDTA, HEDP contains phosphorous instead of nitrogen. In SH, the chlorine essentially carries a positive charge, whereas phosphorous is less electronegative than nitrogen. This could explain the lower reaction rate of HEDP with NaOCl as compared with that of EDTA [31].

A number of studies have reported that a decrease in free available chlorine was accompanied by a decline in pH [31,46,55,56]. Thus, we hypothesised that the pH of the tested irrigants after mixing would correlate with their antibacterial activity. Mixing SH with EDTA and all concentrations of ID resulted in an immediate dramatic drop in pH, while mixing SH with DR18% resulted only in a slight decrease in pH immediately after mixing followed by a more pronounced decrease in pH after 20 min. Pure SH and SH + DR9% and SH + DR15% mixtures maintained an initial pH level during the entire experiment. The effect of HEDP on SH was composition (manufacturer)- and concentration-dependent. Unfortunately, the information on the exact compositions of the tested HEDP formulations is not disclosed by the manufacturers. It may be hypothesised that the ratio of active components (HEDP salt to HEDP acid) differed in the investigated HEDP powders, thus leading to different chemical properties. In our study, higher concentrations of HEDP from the same manufacturer caused a more pronounced decrease in the pH values of SH. The pH values of the irrigant mixtures measured immediately after mixing showed a strong positive correlation with the IZDs for both tested microorganisms; solutions with higher pH values provided greater antibacterial effects. In contrast, some previous studies have reported that a decline in pH may have no influence on the antibacterial effect of SH or may even increase this effect [57,58,59,60,61]. Camps et al. showed that a neutralised SH2.5% solution presented better antibacterial activity against *E. faecalis* than the unmodified SH2.5% solution [59]. Mercade et al. also confirmed that a pH reduction (adjusted to 6.5) in the SH solution improved its anti-*E. faecalis* activity [60]. A review by Rossi-Fedele suggested that reducing the pH value of SH to between 6 and 7.5 would lead to improved action. In our study, SH + ID15% and SH + ID18% with a pH lower than that of the aforementioned provided the lowest antifungal and no anti-*E. faecalis* effects [57].

We readily acknowledge several limitations to our study. First, we used single cultures instead of multispecies bacterial cultures or biofilms. *C. albicans* and *E. faecalis* frequently reside in the root canals as biofilm-forming microorganisms. The resistance of bacteria and fungi in the biofilm to host immune response and antimicrobial agents is considerably higher than that of the planktonic state [62,63,64,65]; therefore, further investigations using endodontic biofilm models are required. Next, we assessed antibacterial activity against only two microorganisms, while endodontic biofilm is a complex multispecies community [66]. Also, we did not assess the effects of the irrigants on the presence of dentin debris. Dentin debris may exhibit an inhibitory effect on the antibacterial properties of different root canal irrigants. Few studies investigated this effect regarding HEDP [67]; therefore, this is an important issue for future research. Finally, EDTA and SH are not intentionally mixed during endodontic treatment; they are used sequentially. Therefore, the effect of EDTA on SH antiseptic activity may be less pronounced. Despite the promising results demonstrated by HEDP in this study, they are limited to in vitro conditions and are difficult to extrapolate to clinical settings. Further studies that will take into account all the aforementioned variables are warranted.

## 5. Conclusions

Within the limitations of the present study, we conclude that most HEDP formulations did not significantly decrease the activity of SH against *C. albicans* and *E. faecalis*. The effect was concentration- and manufacturer-dependent. SH + DR9% was the most effective irrigant against the tested microorganisms.

## Figures and Tables

**Table 1:** The mean diameters of inhibition zones for the tested irrigating solutions.

Irrigating Solution	*C. albicans*		*E. faecalis*	
	Mean (sd)	Median (Q1; Q3)	Mean (sd)	Median (Q1; Q3)
SH	46.4 (2.8) ^a^	45 (45; 47)	12.4 (0.5) ^A^	12 (12; 13)
SH + EDTA	33.8 (0.8) ^b^	34 (33; 34)	12.4 (0.9) ^A^	13 (12; 13)
SH + DR 9%	51.6 (2.1) ^c^	52 (50; 53)	12.4 (0.9) ^A^	12 (12; 12)
SH + DR 15%	44.2 (0.8) ^a^	44 (44; 45)	11.6 (0.5) ^A^	12 (11; 12)
SH + DR 18%	46.8 (3.3) ^a^	48 (45; 49)	12.0 (0.7) ^A^	12 (12; 12)
SH + ID 9%	46.0 (0.7) ^a^	46 (46; 46)	11.6 (0.5) ^A^	12 (11; 12)
SH + ID 15%	23.2 (3.0) ^d^	23 (22; 25)	7.0 (0.7) ^B^	7 (6; 7)
SH + ID 18%	6.6 (0.9) ^e^	7 (6; 7)	6.4 (0.5) ^B^	6 (6; 7)

SH—sodium hypochlorite; EDTA—ethylenediaminetetraacetic acid; DR—dual rinse (etidronate); ID—IsraDent (etidronate); ^A,B^—different uppercase letters in a column indicate statistically significant differences between groups for *E. faecalis*; ^a–e^—different lowercase letters in a column indicate statistically significant differences between groups for *C. albicans*.

## Data Availability

The datasets used and/or analysed during the current study are available from the corresponding author upon reasonable request.
